# A Cyber-Physical Framework for Optimal Coordination of Connected and Automated Vehicles on Multi-Lane Freeways

**DOI:** 10.3390/s23020611

**Published:** 2023-01-05

**Authors:** Yuta Sakaguchi, A. S. M. Bakibillah, Md Abdus Samad Kamal, Kou Yamada

**Affiliations:** 1 Graduate School of Science and Technology, Gunma University, Kiryu 376-8515, Japan; 2 Department of Systems and Control Engineering, School of Engineering, Tokyo Institute of Technology, Tokyo 152-8552, Japan

**Keywords:** cyber-physical framework, connected and automated vehicles, successive optimization, vehicle coordination, vehicle platoon

## Abstract

Uncoordinated driving behavior is one of the main reasons for bottlenecks on freeways. This paper presents a novel cyber-physical framework for optimal coordination of connected and automated vehicles (CAVs) on multi-lane freeways. We consider that all vehicles are connected to a cloud-based computing framework, where a traffic coordination system optimizes the target trajectories of individual vehicles for smooth and safe lane changing or merging. In the proposed framework, the vehicles are coordinated into groups or platoons, and their trajectories are successively optimized in a receding horizon control (RHC) approach. Optimization of the traffic coordination system aims to provide sufficient gaps when a lane change is necessary while minimizing the speed deviation and acceleration of all vehicles. The coordination information is then provided to individual vehicles equipped with local controllers, and each vehicle decides its control acceleration to follow the target trajectories while ensuring a safe distance. Our proposed method guarantees fast optimization and can be used in real-time. The proposed coordination system was evaluated using microscopic traffic simulations and benchmarked with the traditional driving (human-based) system. The results show significant improvement in fuel economy, average velocity, and travel time for various traffic volumes.

## 1. Introduction

Over the past few decades, the primary issues in road transportation systems around the world have been traffic congestion, fuel consumption, greenhouse gas (GHG) emissions, and accidents due to an increase in the number of vehicles and in travel demand [1]. Furthermore, traditional human driving remains to be one of the major causes of traffic bottlenecks, since humans have difficulty accurately anticipating future road traffic conditions and frequently perform acceleration and braking, or instant lane changes. Schrank et al. [2] reported that traffic congestion caused American drivers to spend 8.8 billion extra hours on the road, consuming extra 3.3 billion gallons of fuel, in 2017. Particularly, field studies showed that stop-and-go travel produces 14% more emissions than vehicles traveling at a constant speed [3]. It is evident that effective measures should be taken to reduce the burden of uncontrolled traffic issues for better mobility, fuel economy, and the environment. Therefore, the concept of a coordinated traffic system is currently receiving great interest due to its potential to address a number of issues caused by human drivers, such as stop-and-go driving, travel delays, and traffic accidents [4,5].

The recent developments in connected and automated vehicle (CAV) technologies enable real-time data access and sharing with other vehicles and infrastructure via vehicle-vehicle (V2V), infra-vehicle (I2V), and vehicle-infra (V2I) communications [6,7]. When such necessary information is available, such as states (position, velocity, and acceleration) of other vehicles, destination, and speed limit, it is possible to precisely control the movement and trajectories of individual vehicles to enhance traffic flow efficiency, fuel economy, and driving safety via a connected vehicle environment (CVE) [8,9]. The CVE offers opportunities for effectively realizing better-coordinated traffic in a road network but presents difficulties due to the need to utilize a large amount of data. Additionally, it is feasible to coordinate vehicles utilizing a cloud-based centralized or decentralized controller to enhance traffic flow performance and safety [10,11]. A traffic coordination system can improve traffic flow rate and capacity by making intelligent decisions using guidance information from the global controller. Moreover, the coordination can be repeated to ensure seamless operation even if the vehicle does not execute the command or if unexpected disruptions happen [12]. As a result, it can accurately modify the whole system, which would be very challenging for a human driver to achieve.

A number of studies have developed vehicle coordination systems for CAVs using centralized or decentralized controllers to achieve safe and efficient control of traffic. Some studies developed automated vehicle intersection control systems based on reservation algorithms [13,14,15], whereas some works utilized signal phase and timing (SPAT) information in advance via I2V communication to control the movement of automated vehicles [16,17,18]. Some works proposed optimization of traffic signal phases using the state information (e.g., location and speed) of autonomous vehicles [19,20,21], and some studies developed coordinated intersection control systems for autonomous vehicles under CVE without using traffic signals [22,23,24]. Some works reported coordinated merging control systems for safe and smooth merging of automated vehicles using ramp metering [25,26,27], whereas some other works developed coordinated merging control schemes for efficient merging of CAVs into roundabouts [28,29,30]. These works [13,14,15,16,17,18,19,20,21,22,23,24,25,26,27,28,29,30] mainly focused on vehicle coordination systems for signalized intersections or merging roads.

On the other hand, some works proposed cooperative lane-changing methods for CAVs. For example, Hu et al. [31] and Awal et al. [32] proposed local lane-change coordination of autonomous vehicles, which is limited to local modifications of the traffic. Atagoziyev et al. [33] developed a traffic coordination system for the changing of lanes by autonomous vehicles before reaching a critical position. In each scenario, only one vehicle has the intention to change lanes; the surrounding connective vehicles cooperate together to adjust the formation until the central lane-change vehicle can do so safely; this single-vehicle lane-change process continues sequentially if more than one vehicle intends to change lanes. Li et al. [34] proposed a two-stage multi-vehicle motion planning (MVMP) algorithm for cooperative lane changes of CAVs. After re-configuring a CAV platoon into a sufficiently sparse configuration, all lane changes are carried out simultaneously without attempts to avoid collisions. An and Jung [35] proposed a cooperative lane change protocol considering the impact of V2V communication delay. Although the aforementioned techniques [31,32,33,34,35] can enhance individual driving abilities, they are still inadequate to guarantee smooth lane changes for connected vehicles in congested situations.

In this paper, we develop a novel cyber-physical vehicle coordination system for efficient lane changing or merging of CAVs on multi-lane freeways. To reduce communication volume and computing burden, the vehicles are coordinated into small groups (or platoons), and their trajectories are successively optimized using a receding horizon control (RHC) approach. We assume that the information of CAVs is communicated to a cloud-based computing framework, where an optimization problem is solved to determine target trajectories (speeds and position) of individual vehicles with the goal of providing sufficient gaps during a lane change while minimizing the speed deviation and acceleration of the vehicles. Then, the coordination information is provided to individual vehicles, and the local controller of each vehicle determines its control acceleration to follow the desired trajectories while ensuring driving safety. We have carefully chosen the sizes of vehicle groups, and step and horizon sizes. The model needs less than one second to obtain the optimal solution, and periodically coordinates the vehicles every few seconds to enable the local controller to smoothly control the vehicle in smaller steps toward the target. Therefore, our proposed traffic coordination system guarantees fast optimization and can be implemented in real time. We evaluated the performance of the proposed system using microscopic traffic simulations in view of actual traffic behaviors on a real multi-lane road. It was found that our proposed system significantly improves fuel economy, average velocity, and travel time of vehicles for various traffic volumes compared to traditional human driving.

The paper is organized as follows. Section 2 describes the real traffic scenario and the fundamental idea of our proposed cloud-based vehicle coordination system. Section 3 formulates the optimization problem, including the vehicle driving system and the objective function. Section 4 presents the key simulation results. Finally, Section 5 provides the concluding remarks and future research directions.

## 2. Vehicle Coordination System

### 2.1. Real Scenario

**Figure 1:**
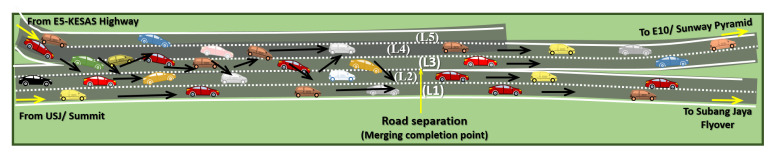
The study route in Subang Jaya, Malaysia: an actual traffic scenario in which vehicles from two roads merge and divert in two ways over a short distance, causing massive congestion every day.

In this paper, we consider a real-world traffic scenario on a real road stretch called Persiaran Kewajipan in Subang Jaya, Malaysia (as shown in Figure 1) to demonstrate the necessity and evaluate the effectiveness of the proposed cloud-based vehicle coordination system. The road segment is multi-lane, and traffic from two roads merges and diverts to both sides over a short distance, causing severe congestion every day. More than half of the vehicles perform multiple lane changes within common sections of about 300 m before diverting onto two distinct routes, and they often struggle to find a safe gap to execute a lane change in such a congested situation. Traffic congestion worsens when a vehicle cannot change lanes efficiently, slows down, or stops other vehicles, causing disruptions in the surrounding traffic and endangering others. It is possible to prevent this sort of traffic congestion by efficiently coordinating all vehicles for timely arrival and lane changes.

### 2.2. Fundamental Idea

**Figure 2:**
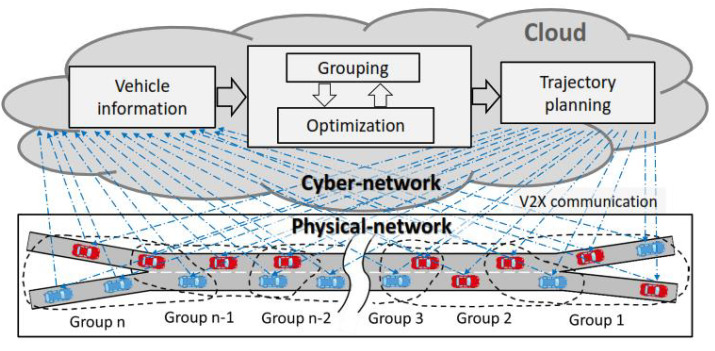
The fundamental idea of our proposed cyber-physical optimal traffic coordinating system. The vehicles are coordinated into groups, and their trajectories are successively optimized.

Figure 2 illustrates the fundamental idea of our proposed traffic coordination system in a cyber-physical framework. It is assumed that every vehicle on the study route is a next-generation CAV that is connected to a cloud or edge computing system, which can perform two-way communications and coordinate vehicles globally with negligible delay. The vehicles transmit their necessary information, such as the current state (position, velocity, and acceleration), the target destination, and other information to the cloud, and the coordination system computes the optimal trajectory of each vehicle. Since it is time-consuming to optimize a large number of vehicles in the cloud due to communication volume and computational burden, for online implementation, the vehicles are coordinated into small groups, and their trajectories are successively optimized. Specifically, vehicles in each group are simultaneously optimized considering the safety constraint imposed by vehicles in the preceding group, and the optimization is repeated in a receding-horizon approach. To maintain maximum traffic performance, the traffic coordination system optimizes vehicle speed and position based on the lane change or merging desires of all relevant vehicles. Based on the coordination information, individual vehicles decide on their acceleration by ensuring smooth and safe lane changes or merging on the freeway.

## 3. Formulation of Optimization Problem

**Figure 3:**
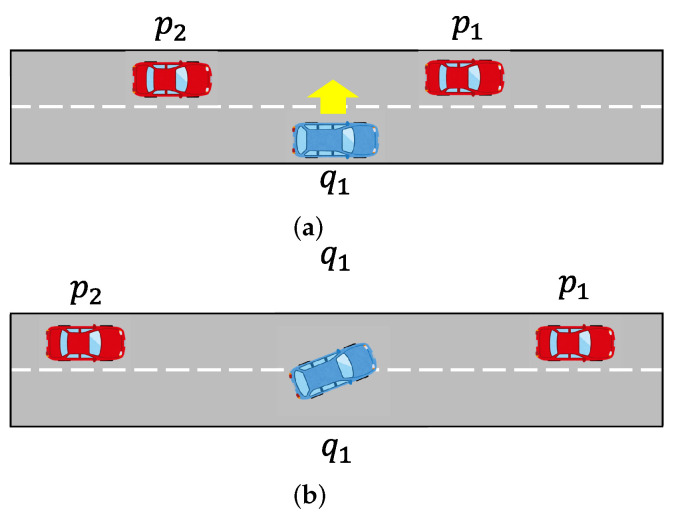
(**a**) A typical scenario for cooperative lane change request, and (**b**) the expected scenario after coordination.

We consider a two-lane freeway where most vehicles require changing lanes, in accordance with the real-world scenario. The coordination method divides all vehicles into groups based on their sequences on the road at regular intervals and successively optimizes each group. Since the optimization is the same for each group of vehicles, we demonstrate the coordination process for one of these groups. In Figure 3a, a scenario with three vehicles is depicted; vehicle $q_{1}$ (in the right lane) needs coordination with vehicles $p_{1}$ and $p_{2}$ (in the left lane) for a smooth lane change. Vehicle $q_{1}$ requests a lane change but cannot change lanes due to the small gap between vehicles $p_{1}$ and $q_{1}$. An example of anticipated solutions is shown in Figure 3b. After receiving the request, vehicles $p_{1}$ and $p_{2}$ adjust their relative distance to allow vehicle $q_{1}$ to change lanes. However, depending on the relative positions and speeds of the vehicles, the expected solutions may differ. Considering traffic performance, a standard rule-based or hierarchical solution may not be effective. Therefore, optimal solutions are desired for all vehicles described below.

### 3.1. Vehicle Driving System

During a trip, a vehicle may change lanes, merge onto a different road, or both, depending on the traffic flow in the lanes and the direction it travels. The state dynamics of any vehicle $n\in N=\{p_{1},p_{2},\ldots ,q_{1},q_{2},\ldots \}$ in either lane (left or right) in discrete time, with a time step *t* of the interval Δt, can be expressed as

(1) $$s_{n}\left(t+1\right)=As_{n}\left(t\right)+Bu_{n}\left(t\right),$$

(2) $$A=\left[\begin{array}{ccc} 1 & \Delta t & 0\\ 0 & 1 & 0\\ 0 & 0 & 1 \end{array}\right],\;\mathrm{and}\;B=\left[\begin{array}{cc} \frac{1}{2}\Delta t^{2} & 0\\ \Delta t & 0\\ 0 & 1 \end{array}\right],$$

where $s_{n}\left(t\right)={\left[x_{n}\left(t\right),v_{n}\left(t\right),\zeta_{n}\left(t\right)\right]}^{T}\in R^{3}$ denotes the state of vehicle *n* in terms of position $x_{n}\left(t\right)$, velocity $v_{n}\left(t\right)$, and current lane $\zeta_{n}\left(t\right)$, respectively; and $u_{n}\left(t\right)={\left[a_{n}\left(t\right),\lambda_{n}\left(t\right)\right]}^{T}\in R^{2}$ is the control vector including acceleration, $a_{n}\left(t\right)\in R$, and the decision about lane changing, $\lambda_{n}\left(t\right)\in \left\{-1,0,1\right\}$, respectively. In this case, $\lambda_{n}\left(t\right)=-1\;\mathrm{or}\;1$ represents a lane change to the left or right, and $\lambda_{n}\left(t\right)=0$ indicates no lane change. Note that the decision to change lanes is constrained by factors related to driving conditions.

Instantaneous acceleration ($a_{n}\left(t\right)$) of vehicle *n* relative to the preceding vehicle n−1 is calculated using a dynamic microscopic car-following model $f_{CF}$ called the intelligent driver model (IDM) [36]:

(3) $$\begin{array}{cc} a_{n}\left(t\right) & =f_{CF}\left(s_{n}\left(t\right),s_{n-1}\left(t\right)\right),\\ \; & =a\left[1-{\left(\frac{v_{n}\left(t\right)}{v_{n}^{d}}\right)}^{4}-{\left(\frac{d^{*}\left(v_{n}\left(t\right),\Delta v_{n}\left(t\right)\right)}{\Delta x_{n}\left(t\right)}\right)}^{2}\right],\\ d^{*}\left(v_{n}\left(t\right),\Delta v_{n}\left(t\right)\right) & =R_{0}+v_{n}\left(t\right)T+\frac{v_{n}\left(t\right),\Delta v_{n}\left(t\right)}{2\sqrt{a_{\max}a_{\min}}}, \end{array}$$

where the parameters $v_{n}^{d}$, $R_{0}$, *T*, $a_{\max}$, and $a_{\min}$ denote the desired speed, minimum gap between vehicles, safe headway time while following the preceding vehicle, maximum acceleration, and comfortable deceleration, respectively; and $\Delta x_{n}\left(t\right)=x_{n-1}\left(t\right)-x_{n}\left(t\right)-l$ (where *l* is the length of the preceding vehicle) and $\Delta v_{n}\left(t\right)=v_{n-1}\left(t\right)-v_{n}\left(t\right)$ are the current distance of the preceding vehicle and the speed difference, respectively. In the framework, the control input at the *t*-th step is updated as $\forall t\in \left[t\Delta t,\left(t+1\right)\Delta t\right],a_{n}\left(t\right)\equiv a_{n}\left(t\Delta t\right)$.

The lane change decision of vehicle *n* depends on the states of some vehicles in the current and target lanes, which can be represented using a well-known lane change model $f_{LC}$ called minimizing overall braking induced by lane change (MOBIL) [37]:

(4) $$\begin{array}{cc} \lambda_{n}\left(t\right) & =f_{LC}\left(s_{n}\left(t\right),s_{n-1}\left(t\right),s_{n+1}\left(t\right),{\bar{s}}_{n-1}\left(t\right),{\bar{s}}_{n+1}\left(t\right)\right),\\ \; & =\left\{\begin{array}{cc} {\tilde{\zeta }}_{n}\left(t\right)-\zeta_{n}\left(t\right), & \mathrm{if}\;\left\{\begin{array}{c} {\tilde{\bar{a}}}_{n+1}\left(t\right)\geq -b_{\mathrm{safe}}\;\mathrm{and},\\ \Delta a_{n}\left(t\right)+\rho \Delta a_{n+1}\left(t\right)\geq \tau , \end{array}\right.\\ 0, & \mathrm{otherwise}, \end{array}\right. \end{array}$$

where ${\bar{s}}_{n-1}\left(t\right)$ and ${\bar{s}}_{n+1}\left(t\right)$, respectively, are the states of the relative preceding and following vehicles in the target lane; ${\tilde{\zeta }}_{n}\left(t\right)\in \left\{\zeta_{n}\left(t\right)+1,\zeta_{n}\left(t\right)-1\right\}$ denotes a lane change from the current lane $\zeta_{n}\left(t\right)$ to the target lane; ${\tilde{\bar{a}}}_{n+1}\left(t\right)$ represents an unsafe lane change of vehicle *n* that may cause aggressive braking of the relative following vehicle ${\bar{a}}_{n+1}$ in the target lane; $-b_{\mathrm{safe}}$ is the safe braking limit; $\Delta a_{n}\left(t\right)$ and $\Delta a_{n+1}\left(t\right)$, respectively, denote an increase in the acceleration of vehicle *n* and a collective increase in the acceleration of the following vehicles in the current and target lanes due to a lane change action; ρ is the politeness factor; and τ is the threshold.

The parameters, ${\tilde{\bar{a}}}_{n+1}\left(t\right)$, $\Delta a_{n}\left(t\right)$, and $\Delta a_{n+1}\left(t\right)$, are calculated using (3) as

$${\tilde{\bar{a}}}_{n+1}\left(t\right)=f_{CF}\left({\bar{s}}_{n+1}\left(t\right),s_{n}\left(t\right)\right),\;\Delta a_{n}\left(t\right)={\tilde{a}}_{n}\left(t\right)-a_{n}\left(t\right),$$

where ${\tilde{a}}_{n}\left(t\right)=f_{CF}\left(s_{n}\left(t\right),{\bar{s}}_{n-1}\left(t\right)\right)$, and

$$\Delta a_{n+1}\left(t\right)=\left({\tilde{a}}_{n+1}\left(t\right)-a_{n+1}\left(t\right)\right)+({\tilde{\bar{a}}}_{n+1}\left(t\right)-{\bar{a}}_{n+1}\left(t\right),$$

where ${\tilde{a}}_{n+1}=f_{CF}\left(s_{n+1}\left(t\right),s_{n-1}\left(t\right)\right)$, $a_{n+1}\left(t\right)=f_{CF}\left(s_{n+1}\left(t\right),s_{n}\left(t\right)\right)$, and

$${\bar{a}}_{n+1}=f_{CF}({\bar{s}}_{n+1}\left(t\right),{\bar{s}}_{n-1}\left(t\right).$$

Based on these parameters, vehicle *n* decides whether to perform a safe lane change or stay at its current lane according to (4). If $\lambda_{n}\left(t\right)=0$, vehicle *n* remains in the current lane $\zeta_{n}\left(t\right)$.

### 3.2. Objective Function

For fuel economy, comfort, and safe driving, sudden acceleration or braking is not beneficial [38]. The proposed cyber-physical traffic coordination system receives information on the states of vehicles within a group or platoon and computes the optimal trajectory for any vehicle *n* using a receding horizon control (RHC) approach. Specifically, we formulate an optimization problem that minimizes an objective function by providing a sufficient gap for smooth and safe lane changing or merging while keeping the speed deviation and acceleration at the optimum level. Some constraints are defined in the optimization that consider driving comfort and regulations related to a road network, such as the speed limit. Moreover, to avoid collisions or aggressive braking, a safe distance between any preceding vehicle n−1 and its following vehicle *n* is necessary, which is dynamically given by the nonlinear constraint as $a_{n}\left(t\right)\leq f_{CF}\left(s_{n}\left(t\right),s_{n-1}\left(t\right)\right)$. This constraint, which blends the optimal action with the naturalistic car-following behavior, ensures a safe gap under any circumstances.

To implement the traffic coordination system, the optimization problem is solved by minimizing an objective function at each time *t* as

(5) $$\begin{array}{cc} J(s_{n}\left(t\right),a_{n}\left(t\right)) & =\sum_{t}^{H}\left\{\sum_{n\in N}\left(w_{1}{\left(v_{n}\left(t\right)-v_{n}^{d}\right)}^{2}+w_{2}a_{n}^{2}\left(t\right)\right)\right.\\ \; & +\sum_{p\in P}\sum_{q\in Q}\left.w_{3}\theta_{pq}\left(t\right)e^{-\alpha {\left(x_{p}\left(t\right)-x_{q}\left(t\right)\right)}^{2}}\right\}, \end{array}$$

subject to

$$\begin{array}{c} v_{\min}\leq v_{n}\left(t\right)\leq v_{\max},\\ a_{\min}\leq a_{n}\left(t\right)\leq a_{\max},\\ a_{n}\left(t\right)\leq f_{CF}\left(s_{n}\left(t\right),s_{n-1}\left(t\right)\right),\\ \theta_{pq}\left(t\right)=\left\{\begin{array}{cc} 0, & \mathrm{if}\;\delta_{p}\left(t\right)+\delta_{q}\left(t\right)=0,\\ 1, & \mathrm{otherwise}. \end{array}\right. \end{array}$$

where *H* is the time horizon; N is the set of vehicles in the partial optimization group; P and Q are the numbers of vehicles in the left and right lanes, $\theta_{pq}\in \left\{0,1\right\}$ is a binary variable to enable the third cost term in the objective function and depends on $\delta_{p},\delta_{q}\in \left\{0,1\right\}$, which denotes the need for the vehicles to change lanes (i.e., 1 indicates a lane change is necessary and vice versa); α is a positive constant; $x_{p}$ and $x_{q}$ are the positions of vehicles in the left and right lanes; and $w_{1}$, $w_{2}$, and $w_{3}$ are the weighting factors corresponding to the velocity, acceleration, and safe lane change terms, respectively. The first term of the objective function implies a penalty when the current velocity $v_{n}\left(t\right)$ of vehicle *n* deviates from $v_{n}^{d}$, the second term is the cost of acceleration along the freeway, and the third term represents a penalty for an unsafe lane change at the target time *t* due to an insufficient gap.

Note that the weights $w_{1}$ and $w_{2}$ balance the squares of the speed deviation and acceleration costs into a single value in this composite single objective optimization. Usually, the square of speed deviation can be very large compared to the square of acceleration (with their typical ranges). Hence, to emphasize the influence of both in (5), $w_{1}$ needs to be smaller than $w_{2}$ (depending on the maximum values of each cost term). However, we further tuned $w_{1}$ and $w_{2}$ based on trials and performance observations, which is a common practice in similar single-objective optimization tasks. On the other hand, $w_{3}$ is set to a high value to ensure that the safety cost is dominant when a lane change gap is necessary. The objective function *J* is minimized by selecting the proper speed for each vehicle *n*, subject to the aforementioned constraints. We assume that the states and destinations (target lane) of all vehicles are available to the cloud-based computing framework, where the optimization problem is solved for successive groups of vehicles, and the optimized target speeds and positions are subsequently communicated to individual vehicles. After obtaining the coordination information, the lane change of each vehicle is implemented using (4). In such a manner, the controller drives the vehicles safely until the next coordination phase, and the optimization is repeated in the cloud for the new group of vehicles.

In this paper, the optimization aims to periodically coordinate vehicles to create sufficient gaps for lane-changing vehicles, which is possible with a larger step size, since the actual vehicle control is performed with a short step size to ensure safety and smooth maneuvering. For any optimization scheme, the problem size increases with the number of vehicles involved and the horizon length, resulting in a costly optimal solution that is often impractical to apply in real-time. Here, we focus on the total computational costs with the necessary communication volume to keep it manageable for the implementation of the cyber-physical framework. We consider small vehicle groups to reduce the computational burden of coordinating them successively. However, with a long horizon, the interaction between the consecutive small groups becomes complicated due to many lane-change actions of vehicles on the divided road ahead over a short distance. With some sensitivity observations, we have manually tuned the vehicle group size, time step size, and horizon length and come to the settings considered in this paper. Note that the horizon length needs to be tuned similarly for different group sizes and road contexts.

## 4. Simulation Results

To demonstrate the effectiveness of the proposed traffic coordination system, we developed a multi-lane simulation framework in MATLAB (which has been demonstrated to be mathematically reliable and utilized to model numerous real-world situations) based on the real study route and solved a nonlinear optimization problem (described in (5)) in discrete time. The arrival of vehicles in the simulator was decided randomly using a probability distribution to produce realistic traffic flows. In the simulation, all vehicles were considered to be of the same size and length. The simulation parameters were chosen as $v_{n}^{d}=23$ m/s, $R_{0}=2$ m, T=1.5 s, l=5 m, $v_{n}\in \left[0,25\right]$ m/s, $a_{n}\in \left[-2.5,1.5\right]$ m/s^2^, $b_{\mathrm{safe}}=5$ m/s^2^, ρ=0, τ=0.25, $w_{1}=0.1$, $w_{2}=1$, $w_{3}=0.3$, and α=0.001. We set a suitable prediction horizon of H=5 s with 10 steps and the step size of Δt=0.5 s. Note that ρ varies among drivers depending on the driving contexts and behavior. For realistic behavior in the discretionary lane change, the typical values of ρ can be between 0.2 to 0.5; however, in our context, it is a mandatory lane change, and thus, ρ was set to 0; i.e., a lane change was executed only when the safety criteria were satisfied.

**Figure 4:**
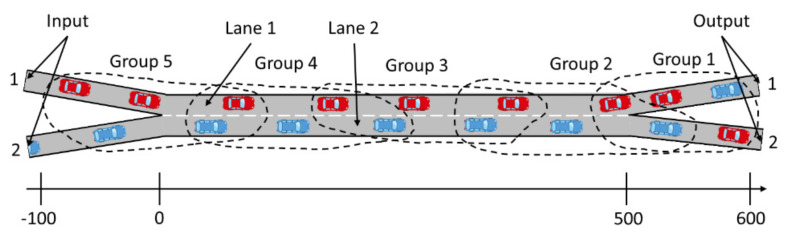
Multi-lane road network used for simulation and evaluation of the proposed traffic coordination system.

The simulation framework is depicted in Figure 4, whereby roadways 1 and 2 share a portion between 0 and 500 m, where vehicles may change lanes depending on their destinations. In traditional driving systems, vehicles that need to change lanes slow down and wait for the appropriate time to do so when approaching the merging junction. Consequently, the vehicles in the opposite lane may also slow down to make space for the awaiting vehicles to change lanes, which may reduce the overall traffic flow performance in the network. Moreover, finding safe gaps to perform lane changes in a congested situation is challenging. In the proposed traffic coordination system, vehicles between −100 and 600 m are divided into multiple groups and optimized every 5 s. Note that here we consider a suitable optimization horizon; since traffic flow experiences substantial variations, a long horizon would not be helpful.

**Figure 5:**
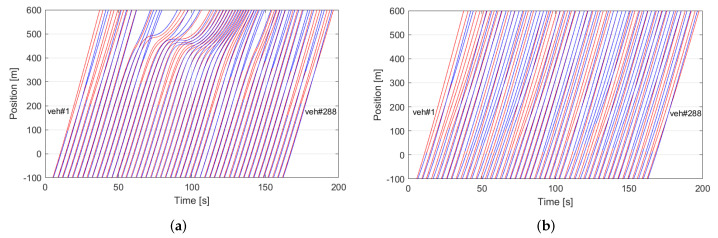
Trajectories of the vehicles traveling about 600 m in 200 s on the model multi-lane freeway. The sub-figures show (**a**) the traditional driving system and (**b**) the proposed traffic coordination system.

**Figure 6:**
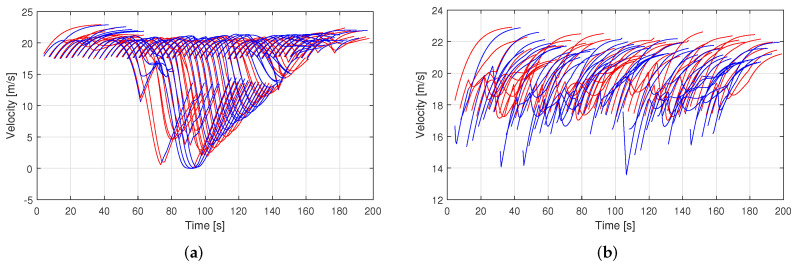
Velocity profiles of the vehicles showing speeding and slowing down characteristics for (**a**) the traditional driving system and (**b**) the proposed traffic coordination system.

**Figure 7:**
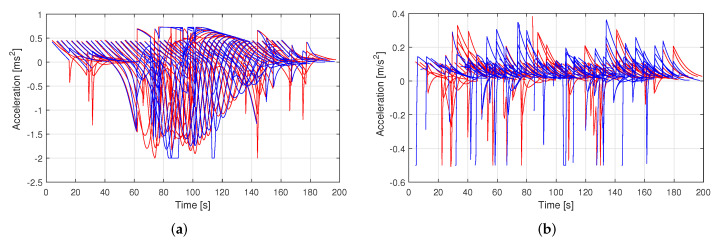
Acceleration profiles of the vehicles showing the level of aggressiveness for (**a**) the traditional driving system and (**b**) the proposed traffic coordination system.

Figure 5 shows the trajectory of each vehicle in dense traffic for both traditional and coordinated driving systems while traveling about 600 m on the studied multi-lane freeway. In the traditional driving system (Figure 5a), some vehicles are unable to change lanes in time, slowing them down and blocking others, causing long queues and traffic congestion, whereas in the proposed coordination system (Figure 5b), vehicles can smoothly change lanes without interrupting surrounding traffic. The velocity profiles of the vehicles for the traditional driving system and the proposed coordination system are shown in Figure 6a,b, respectively. In the traditional driving system, some vehicles quickly slow down and/or come to a complete stop before lane changes. In the proposed coordination system, however, vehicles can smoothly change lanes by slowing down from the peak speed to a level of about 14 m/s. Figure 7 depicts the acceleration profiles of these vehicles. Compared to the traditional driving system, the proposed coordination system performs significantly less deceleration of about −0.4 m/s^2^ during lane changes. The aggressive braking in traditional driving reduces traffic performance and driving safety. In contrast, smooth braking in coordinated driving promotes improved kinetic energy usage, which lowers vehicle fuel consumption.

**Figure 8:**
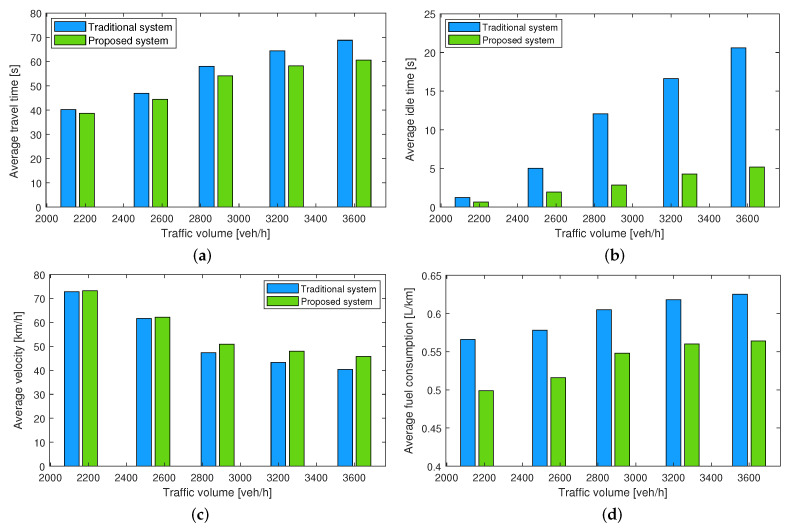
Performance comparison (**a**) average travel time, (**b**) average idling time, (**c**) average velocity, and (**d**) average fuel consumption of the traditional driving system and the proposed traffic coordination system for various traffic volumes on the model freeway.

Figure 8 compares the simulation results of the traditional driving system and the proposed traffic coordination system using four important performance measures of traffic flow, including average travel time, average idling time, average velocity, and average fuel consumption. The traveling time is the time it takes for a vehicle to drive the study road segment, and the idling time is the total time it takes for a vehicle to stop and wait at the merging junction during lane changing. The average speed is the total speed of all vehicles divided by the total number of vehicles in the simulation. The average fuel consumption is the cumulative fuel consumption divided by the number of vehicles in the road segment. In the paper, the fuel consumption of vehicles is determined based on trajectory data (instantaneous speed and acceleration) of vehicles using the VT-Micro model [39]. The model was experimentally developed at Oak Ridge National Laboratory (ORNL) with nine regular-emitting light-duty vehicles. Using chassis dynamometer data collected at the ORNL, different polynomial combinations of acceleration and velocity were investigated using this model. The model is well-accepted and widely used in transportation studies to determine the fuel consumption of vehicles.

Figure 8a,b, respectively, show that the average travel time and the idling time in the proposed traffic coordination system are significantly reduced compared to those in the traditional driving system for various traffic levels. This is due to the fact that the coordinated vehicles make early decisions and require minimal waiting time to execute lane changes. Furthermore, the proposed coordination system considerably increases the average speed compared to the traditional system (as shown in Figure 8c) because coordinated vehicles rarely need to slow down or stop before changing lanes or merging, resulting in smoother flow. Finally, Figure 8d illustrates the comparison of average fuel consumption for both systems. It is evident that the proposed traffic coordination system outperforms the traditional driving system for various volumes of traffic. The percentage improvements in the average travel time, the average velocity, and the average fuel consumption by the proposed traffic coordination system are given in Table 1.

## 5. Conclusions

In this paper, we have developed a novel cyber-physical framework for optimal coordination of CAVs on multi-lane freeways. Using a receding horizon control (RHC) approach, the vehicles are coordinated into successive groups for a smooth and safe lane change or merging. We assume that the information of all vehicles is available to a cloud-based computing framework, where an optimization problem is solved to calculate the target speeds and positions of individual vehicles in the groups. Following that, the coordination information is provided to individual vehicles, and the local controller of each vehicle determines its control acceleration in order to follow the desired trajectory while ensuring driving safety. The proposed traffic coordination system was evaluated considering real-world traffic conditions on a real multi-lane road. The results show that the proposed framework significantly improves the fuel consumption, average velocity, and travel time for vehicles in various amounts of traffic. Our proposed method can be implemented online, as the computing burden is almost negligible.

In future work, we will investigate mixed traffic performances for various penetration rates of CAVs. The proposed framework can be further extended using distributed model predictive control (MPC) for individual vehicles.

## Figures and Tables

**Table 1:** Performance comparison between the proposed traffic coordination system and the traditional driving system.

	Traditional System	Coordination System	Improvement
Average travel time [s]	55.68	51.20	8.05%
Average velocity [km/h]	53.08	56.02	5.53%
Average fuel consumption [L/km]	0.5984	0.5374	10.19%

## Data Availability

Not applicable.
